# Reflectance confocal microscopy of spiradenoma

**DOI:** 10.1016/j.jdcr.2022.04.027

**Published:** 2022-05-06

**Authors:** Pedro Lobos, Constanza Lobos, Karol Baksai

**Affiliations:** a Department of Dermatology, Clínica Las Condes, Santiago, Chile; b Department of Dermatology, Universidad de La República, Montevideo, Uruguay; c Department of Pathology, Clínica Las Condes, Santiago, Chile

**Keywords:** reflectance confocal microscopy, spiradenoma, RCM, reflectance confocal microscopy

## Clinical presentation

**Fig 1:**
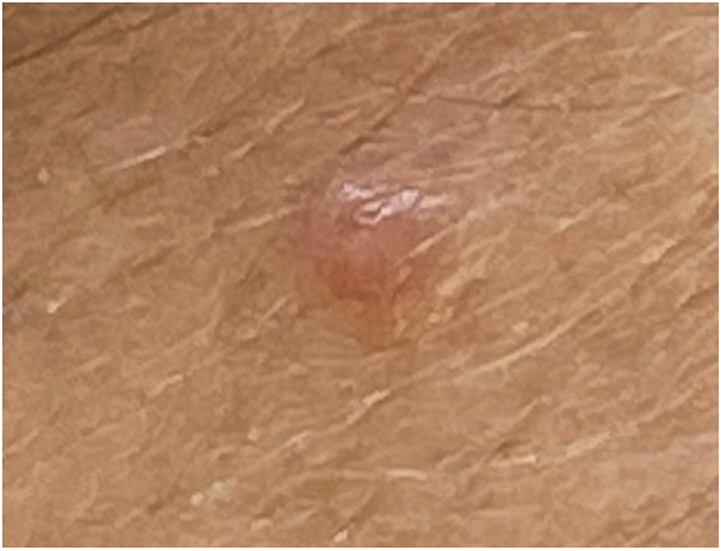
A well-circumscribed, 4-mm, skin-colored papule was noticed on the right portion of the patient’s forehead.

A 69-year old woman presented to Dermatology for a skin cancer screening. Clinical examination found a 4-mm skin-colored papule on her forehead. The lesion had been present for 3 years, was asymptomatic, and had remained unchanged (Fig 1).

### Dermatoscopy

**Fig 2:**
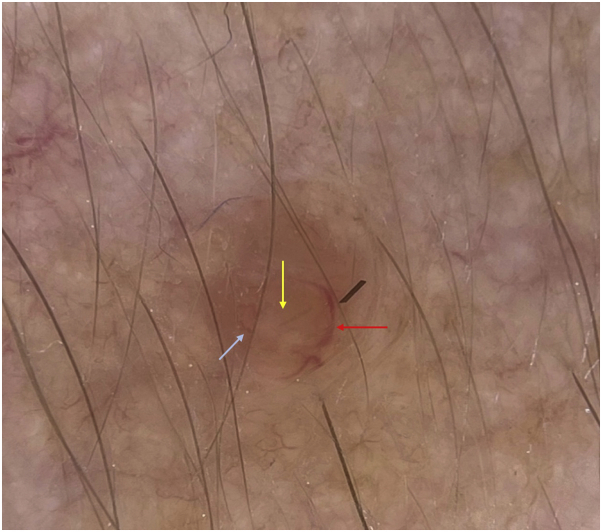
Dermatoscopy showed a homogeneous, brown-orange–colored papule (*yellow arrow*) with linear vessels at the periphery of the lesion (*red arrow*) and out-of-focus, short telangiectasia (*light-blue arrow*).

**Fig 3:**
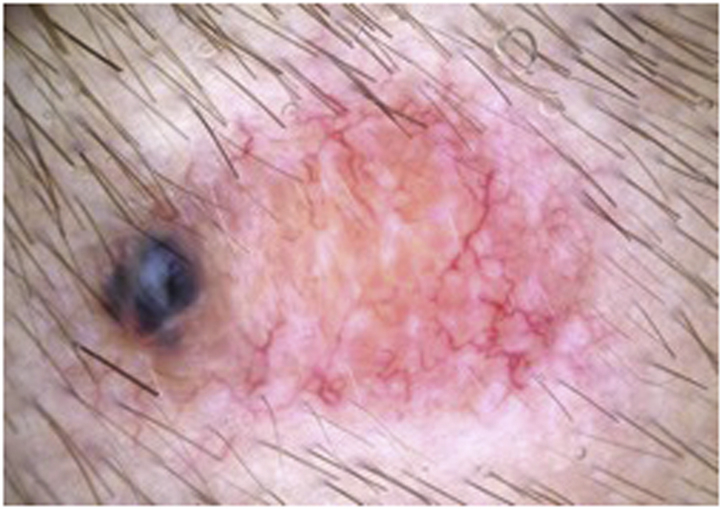
Dermatoscopic pattern of a spiradenoma: blue and orange clods as well as branched vessels can be observed. [Copyright: ©2012 Tschandl.]^1^

Dermatoscopy revealed a well-circumscribed homogeneous brown-orange color papule with linear vessels at the periphery of the lesion that were out-of-focus (Fig 2) in contrast with other findings published in the literature (Fig 3).^1^

### Reflectance confocal microscopy

**Fig 4:**
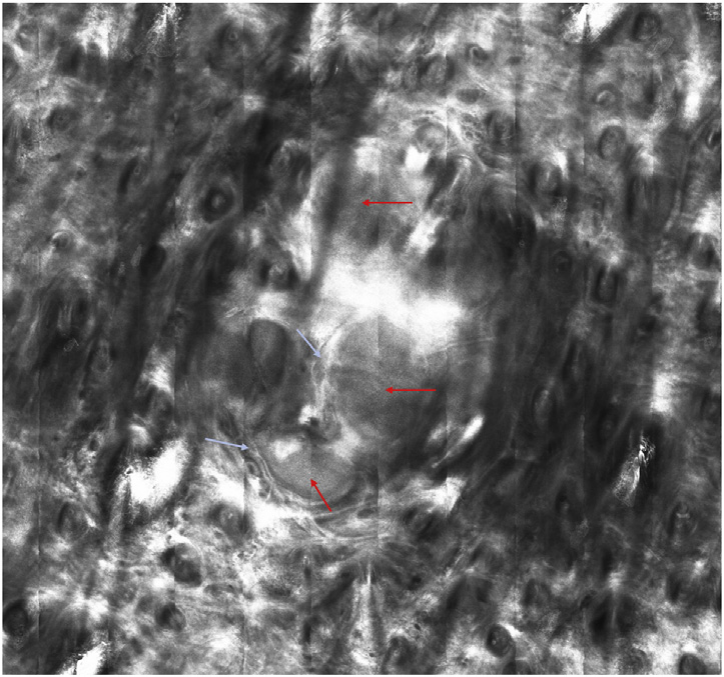
RCM showed a multilobulated, central lesion with a regular, white dot pattern present within the tumor islands (*red arrows*) that were surrounded by bright collagen bundles (*light-blue arrows*).

**Fig 5:**
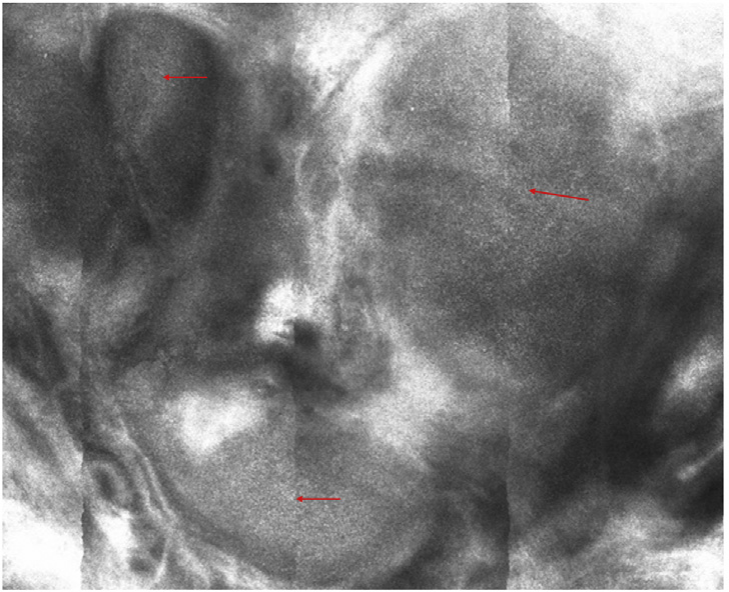
*Red arrows* showing the homogeneous, white dot pattern within the tumor islands.

**Fig 6:**
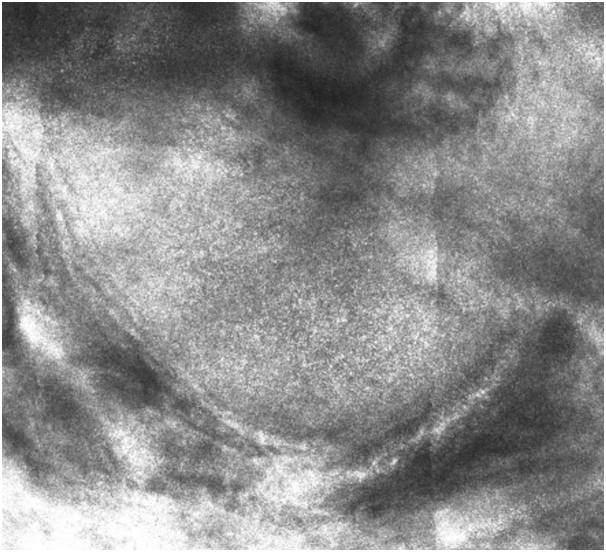
Close-up of a tumor island revealing details of the homogeneous, white dot pattern.

Reflectance confocal microscopy (RCM) of the reticular dermis showed a central area with rounded dermal nodules of different sizes with a white dot pattern, surrounded by bright collagen bundles, with no plump-bright cells outside the tumor area. Palisading and peripheral dark, cleft-like spaces, which are typical diagnostic features of basal cell carcinoma, were absent (Fig 4, Fig 5, Fig 6).

### Histopathology

**Fig 7:**
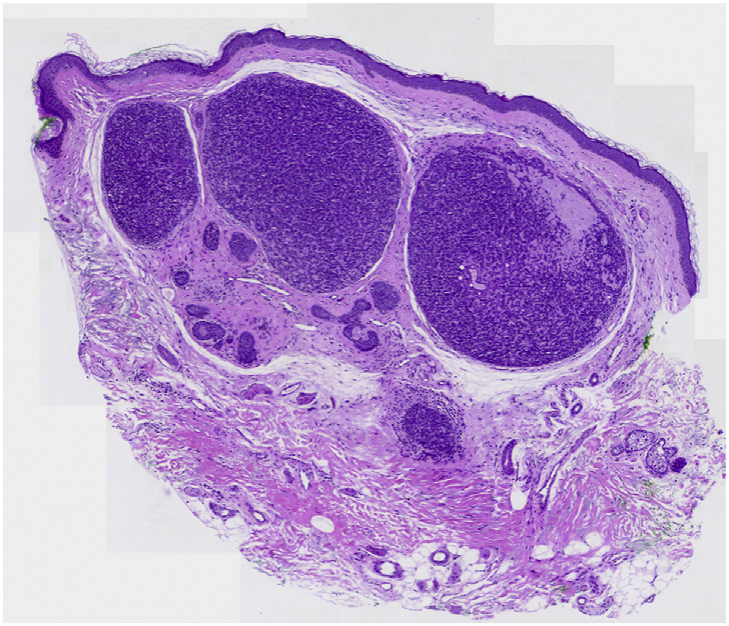
A typical histopathologic pattern of spiradenoma with blue basaloid cells in a layout of eosinophilic basement-membrane material (H&E stain 20×).

**Fig 8:**
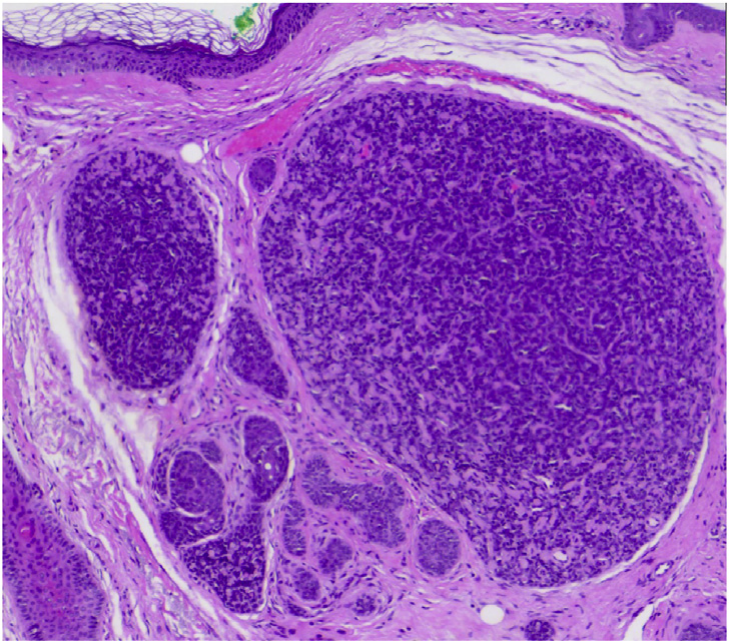
Details of the blue basaloid cells in a layout of eosinophilic basement-membrane material (H&E stain 20× close-up).

Histopathology showed photodamaged skin with sharply demarcated basophilic nodules in the dermis, composed of basaloid cells with a small hyperchromatic nucleus and scant cytoplasm and some larger pale cells with an ovoid nucleus. The cells were surrounding globules of eosinophilic basement-membrane material. Moreover, duct-like structures and some cystic cavities with eosinophilic material could be identified (Figs 7 and 8).

## Discussion

Spiradenomas are uncommon, well-differentiated, benign, dermal neoplasms originating in sweat glands. They typically appear as small solitary nodules, except when they are found in Brooke-Spiegler syndrome, in which case they are multiple and occur with other adnexal neoplasias.^2^ They usually develop on the head, neck, and trunk and are strikingly painful. Their dermatoscopic features have been previously described^1^^,^^3^^,^^4^ and are characterized by telangiectasia with branched vessels. Occasionally, they exhibit blue clods and serpentine vessels. Our case was distinct, because no blue pigment was found, and only linear vessels were observed. The RCM revealed a dermal multilobulated skin neoplasm with a homogeneous, white dot pattern within the tumoral nodules that differed from sebaceous hyperplasia, in which the RCM findings typically reveal enlarged sebaceous lobules around a central, dark round structure that correlates with the dilated sebaceous duct and morule-like structures formed by a group of cuboidal cells with centrally located, dark nuclei and bright, speckled cytoplasm corresponding to the sebaceous cells.^5^ We postulate that the homogeneous, white dot pattern is caused by the collagen present in the globules of eosinophilic basement-membrane material, as this structure was highly refractile. No RCM signs of basal cell carcinoma were observed; namely, bright tumor islands or dark silhouettes, cleft-like, dark spaces, dendritic cells, or plump-bright cells infiltrating the tumor islands or in the periphery. To our knowledge, this is the first reported case of RCM findings in spiradenoma. Finally, we suggest, although more data are necessary to confirm our findings, that a spiradenoma should be suspected, when the RCM evaluation of a skin tumor reveals: (1) a multilobulated, well-circumscribed dermal tumor; (2) a typical homogenous white dot pattern within the tumoral nodules; and (3) an absence of RCM-based signs of basal cell carcinoma.

## Conflicts of interest

None disclosed.
